# Switching between Three Types of Mesalazine Formulation and Sulfasalazine in Patients with Active Ulcerative Colitis Who Have Already Received High-Dose Treatment with These Agents

**DOI:** 10.3390/jcm8122109

**Published:** 2019-12-02

**Authors:** Eriko Yasutomi, Sakiko Hiraoka, Shumpei Yamamoto, Shohei Oka, Mami Hirai, Yasushi Yamasaki, Toshihiro Inokuchi, Hideaki Kinugasa, Masahiro Takahara, Keita Harada, Jun Kato, Hiroyuki Okada

**Affiliations:** 1 Department of Gastroenterology and Hepatology, Okayama University Graduate School of Medicine, Dentistry and Pharmaceutical Sciences, Okayama 700-8558, Japan; eriko.yasutomi.piano@gmail.com (E.Y.); ysyunpei@hotmail.com (S.Y.); oka.shouhei@gmail.com (S.O.); mami_k@live.jp (M.H.); yasshifive@yahoo.co.jp (Y.Y.); toshpi@yahoo.co.jp (T.I.); gyacy14@yahoo.co.jp (H.K.); m_takahara009@yahoo.co.jp (M.T.); keita818@cc.okayama-u.ac.jp (K.H.); hiro@md.okayama-u.ac.jp (H.O.); 2 Department of Gastroenterology, Graduate School of Medicine, Chiba University, Chiba 260-0856, Japan; kato.jun@chiba-u.jp

**Keywords:** ulcerative colitis, salicylates, mesalazine, sulfasalazine

## Abstract

Background and aim: Oral mesalazine and sulfasalazine (SASP) are key drugs for treating ulcerative colitis (UC). The efficacy of switching from one of the several mesalazine formulations to another is largely unknown. This study assessed the efficacy of switching among three types of mesalazine formulation and SASP for UC therapy. Methods: UC patients receiving high-dose mesalazine/SASP who switched to other formulations due to disease activity were considered eligible. Efficacy was evaluated 2, 6, and 12 months after switching. Results: A total of 106 switches in 88 UC patients were analyzed. The efficacy at 2 months after switching was observed in 23/39 (59%) cases from any mesalazine formulation to SASP, in 18/55 (33%) cases from one mesalazine to another, and in 2/12 (17%) cases from SASP to any mesalazine formulation. Nine of 43 effective cases showed inefficacy or became intolerant post-switching. Delayed efficacy more than two months after switching was observed in four cases. Steroid-free remission was achieved in 42/106 (39%) cases—within 100 days in 35 of these cases (83%). Conclusions: Switching from mesalazine to SASP was effective in more than half of cases. The efficacy of switching between mesalazine formulations was lower but may be worth attempting in clinical practice from a safety perspective.

## 1. Introduction

Ulcerative colitis (UC) is a chronic idiopathic disorder characterized by manifestations such as rectal bleeding, diarrhea, abdominal pain, a fever, anemia, and loss of body weight. [1]. Several etiologies of this disease have been found, but it is still difficult to cure completely. Therefore, better disease control with continuation of adequate therapies is important. There are various medications for UC, such as 5-aminosalicylic acids, thiopurines, corticosteroids, calcineurin inhibitors, anti-tumor necrosis factor (TNFα) antibodies, anti-α4β7integrin antibodies, and Janus kinase inhibitors [2,3]. These medications, however, are not always effective in UC patients, and some show a loss of response despite initial efficacy. In this regard, physicians must administer these agents carefully to UC patients. 

Oral mesalazine and sulfasalazine (SASP) are first-choice drugs for treating mild-to moderate UC. In addition, these agents are useful as both induction therapy and maintenance therapy [4,5] and show sufficient safety with long-term usage. In the early days of UC treatment, only SASP was available. SASP consists of a mesalazine and a sulfapyridine molecule bound by an azo bond, which is cleaved upon exposure to colonic bacteria. Systemic absorption of sulfapyridine induces many of the adverse effects associated with sulfasalazine [6]. 

In this situation, new formulations containing only the mesalazine component have been developed. However, uncoated mesalazine is readily absorbed in the upper jejunum and unable to reach the colon in therapeutic concentrations [7,8]. Studies on alternative mesalazine delivery systems have consequently resulted in several formulations of oral mesalazine being developed. In Japan, for example, three types of mesalazine formulation have been approved for treatment of UC: time-dependent mesalazine (Pentasa), pH-dependent mesalazine (Asacol), and once-daily multi-matrix system (MMX) mesalazine (Lialda).

Comparisons of the efficacy among these three formulations have been reported. However, studies with fair comparisons are scarce because the doses of comparative mesalazine formulations are sometimes different (e.g., 2.25 g/day of Pentasa vs. 3.6 g/day of Asacol). Notably, in the few studies using similar doses of different types of mesalazine formulation (3 g/day of pH-dependent mesalazine vs. 3 g/day of time-dependent mesalazine [9] and 2.4 g/day of MMX mesalazine vs. 2.25 g/day of time-dependent mesalazine [10]), an equivalent efficacy and safety were observed in mild to moderately active UC patients. 

In addition, comparisons between mesalazine and SASP have also been reported. However, the equivalent doses between mesalazine and SASP are uncertain, so the fairness of those comparisons is unclear. Furthermore, the efficacy of switching between mesalazine formulations and SASP has scarcely been reported. Yoshino et al. [11] reported the efficacy of SASP treatment for UC patients who relapsed despite the oral administration of >4 g/day time-dependent mesalazine or 3.6 g/day of pH-dependent mesalazine and showed that 69.4% of 36 refractory-UC patients achieved clinical remission after initiating treatment with SASP. However, few reports have described the efficacy of switching from one mesalazine formulation to another. 

The efficacy of switching between high-dose mesalazine/SASP is a clinical question with great interest in real clinical practice, as patients in whom high-dose mesalazine/SASP is insufficiently effective may be switched to immunosuppressive agents or biologics that are likely to be accompanied by adverse events and a high expense. However, reports regarding this issue are scarce. 

Therefore, we investigated the short- and long-term efficacy of switching among mesalazine/SASP formulations in UC patients who had already received high-dose mesalazine/SASP. 

## 2. Methods

### 2.1. Patients

Eligible subjects were UC patients who had switched oral mesalazine/SASP formulations at Okayama University Hospital between January 2006 and March 2019 and had been followed for >12 months after the switch. Patients who had received the highest dose of mesalazine formation or SASP approved in Japan (4 g/day for time-dependent mesalazine (Pentasa), 3.6 g/day for pH-dependent mesalazine (Asacol), and 4.8 g/day for once-daily MMX mesalazine(Lialda)) were included. Patients who had been administered ≥3 g SASP/day were also considered eligible. 

However, cases with switching of mesalazine/SASP due to reasons other than therapy intensification (e.g., adverse events or the patient’s request) were not included. In addition, cases starting other therapies within a certain period before the switching were also excluded. The intervals allowed before switching were more than two weeks for topical mesalazine, corticosteroids, anti-TNF agents, tacrolimus, or cytapheresis and more than four weeks for immunomodulators (azathioprine/mercaptopurine). 

In patients who experienced multiple episodes of mesalazine/SASP switching, each episode was included if the evaluation of the prior episode was finished. Therefore, 16 patients with 2 episodes were included, as was 1 patient with 3 episodes.

The choice of formulation at the switch depended on each physician’s preference. In principle, SASP was considered when high-dose mesalazine was insufficient. However, SASP was contraindicated in a considerable number of patients due to their history or concern regarding adverse effects (e.g., male infertility); thus, switching between mesalazine formulations was relatively frequent. For switching between mesalazine formulations or from SASP to mesalazine, the prescription of the maximum dose of one formulation was immediately changed to a prescription of the maximum dose of another formulation. For switching from mesalazine to SASP, the dose of SASP was gradually increased with the gradual decrease in the dose of mesalazine over a period of up to six weeks if adverse events associated with SASP, such as headache and nausea, were of concern. In principle, patients who underwent switching received a high dose of mesalazine or SASP for 12 months or longer after the switch, because these patients were considered to be likely to relapse if the doses of these agents were reduced, even after an effective switch.

### 2.2. Evaluated Outcomes

The efficacy was evaluated at 2, 6, and 12 months after switching. The clinical activity was evaluated using a two-item patient-reported outcome (PRO2), consisting of a rectal bleeding subscore (RBS) and stool frequency subscore (SFS), [12] extracted from the Mayo score [13]. The clinical courses were classified as remission, improvement, and inefficacy. The definitions were as follows: remission occurred when both the RBS and SFS were 0, improvement occurred when the RBS and/or SFS had decreased but not reached remission, and inefficacy occurred when neither remission nor improvement had been achieved. Both remission and improvement were taken to indicate “efficacy.” In addition, the addition of other therapies or escalation of concomitant drugs prior to the evaluation time points was considered to indicate “inefficacy.” The reduction of concomitant drugs in association with symptom improvement was allowable. The PRO2 results were confirmed by another activity index (Lichtiger clinical activity index [14], Figure S1).

Steroid-free remission was defined as an achievement of steroid-free remission after the switching of mesalazine formulations without additional therapy. A quantitative fecal immunochemical test (FIT) was performed at each patient visit as a marker of mucosal healing. A negative FIT result (<100 ng/mL) can predict mucosal healing (Mayo endoscopic score 0) with 92–95% sensitivity and 62–71% specificity [15,16,17]. The FIT was performed with the OC-Sensor DIANA (Eiken Chemical, Tokyo, Japan).

This single-center retrospective observational study was approved by the institutional review board of Okayama University Graduate School of Medicine, Dentistry, and Pharmaceutical Sciences, and there were no conflicts of interest or sponsors of this study (IRB approval number: 1806-036).

### 2.3. Statistical Analyses

The efficacy of switching formulations was compared using Fisher’s exact test. *p*-values < 0.05 were considered statistically significant. The times to steroid-free remission were analyzed via the Kaplan–Meier method. The statistical analyses were performed using the JMP pro software program, ver. 14 (SAS Institute, Cary, NC, USA).

## 3. Results

### 3.1. Patient Characteristics

**Figure 1:**
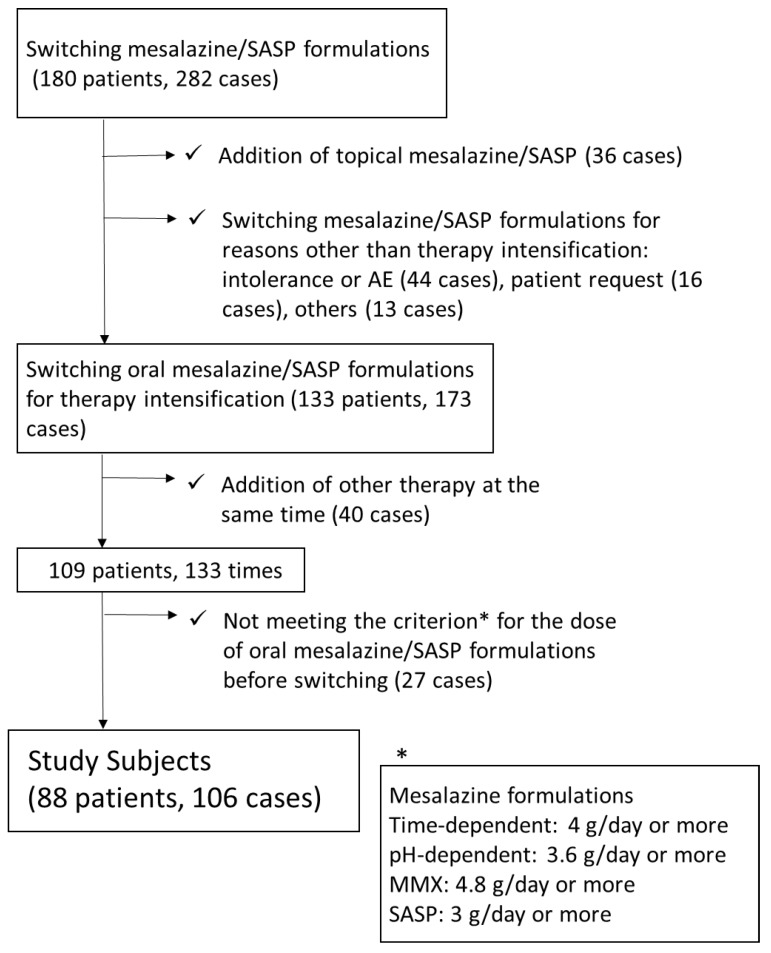
Flow diagram of the study. *Criterion for the dose of oral mesalazine/SASP formulations before switching. SASP: ≥3 g/day; time-dependent mesalazine: 4 g/day; pH-dependent mesalazine: 3.6 g/day; once-daily multi-matrix system (MMX) mesalazine: 4.8 g/day. SASP: sulfasalazine.

**Figure 2:**
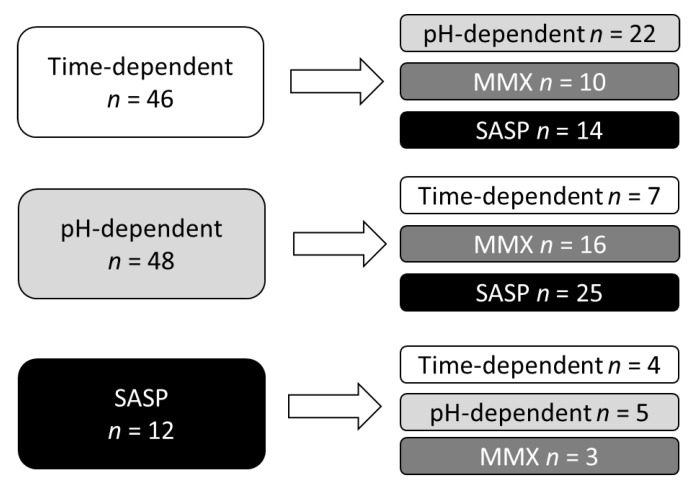
Details of mesalazine formulations/SASP before and after switching.

The data of 282 switching cases in 180 UC patients were collected, and 106 cases in 88 UC patents were eligible for our analysis (Figure 1). The clinical characteristics of the analyzed patients are summarized in Table 1. The median PRO2 was 2, suggesting that the disease activity of switching candidates was not very high. Figure 2 shows the details of the mesalazine formulations/SASP before and after switching. Before switching, time-dependent mesalazine, pH-dependent mesalazine, and SASP were prescribed to 46, 48, and 12 patients, respectively. No patients were administered once-daily MMX mesalazine before switching. After switching, the numbers of patients with time-dependent mesalazine, pH-dependent mesalazine, once-daily MMX mesalazine, and SASP were 11, 27, 29, and 39, respectively. 

### 3.2. Short-Term Efficacy of Switching

**Figure 3:**
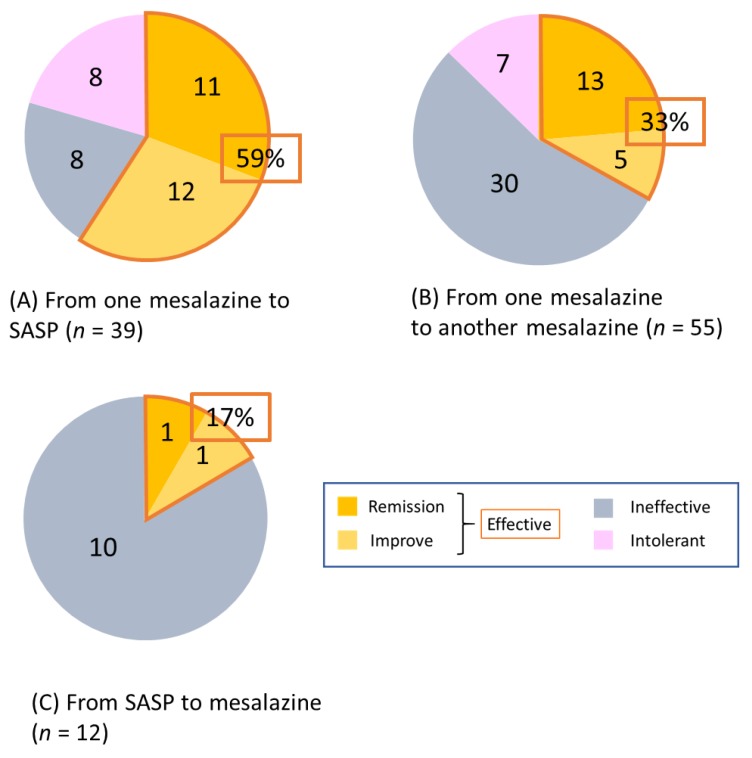
Effectiveness at two months after switching mesalazine/SASP formulations. (**A**) from any mesalazine to SASP (*n* = 39), (**B**) from one mesalazine to another (*n* = 55), and (**C**) from SASP to any mesalazine (*n* = 12). SASP: sulfasalazine.

The short-term efficacy was evaluated two months after switching. Of the 39 cases of switching from any mesalazine formulation to SASP, efficacy was observed in 59% of cases (23/39), which included 11 (28%) cases in remission and 12 (31%) cases with improvement. Intolerance to SASP was observed in 8 (20%) cases (Figure 3A).

Of the 55 cases of switching from any mesalazine formulation to another, efficacy was observed in 33% of cases (18/55), which included 13 (23%) cases in remission and 5 (9%) cases with improvement. Two-thirds of those switching showed inefficacy or intolerance (Figure 3B).

Of the 12 cases of switching from SASP to mesalazine, efficacy was observed in only 2 (17%). All remaining cases showed inefficacy, with no intolerance observed (Figure 3C). The efficacy of switching from mesalazine to SASP was significantly higher than that of the other types of switching (mesalazine to mesalazine or SASP to mesalazine) (*p* = 0.014). The results of the analysis based on each mesalazine formulation are shown in Figure S2.

### 3.3. Long-Term Outcomes of Switching 

**Figure 4:**
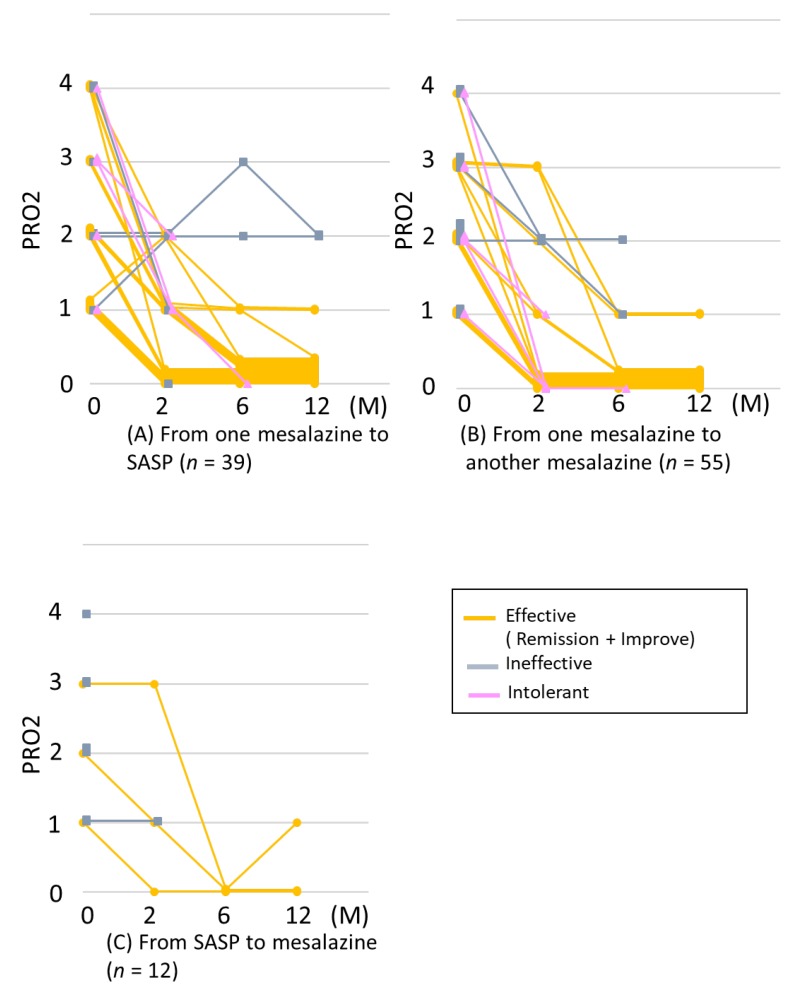
Courses of PRO2 after switching mesalazine/SASP formulations. (**A**) from any mesalazine to SASP (*n* = 39), (**B**) from one mesalazine to another (*n* = 55), and (**C**) from SASP to any mesalazine (*n* = 12). If any other therapies were added or if mesalazine was used after switching was stopped, the graph lines were censored. SASP: sulfasalazine.

We examined the changes in the PRO2 at 2, 6, and 12 months after switching (Figure 4). Of the 23 cases of efficacy at 2 months after switching from mesalazine to SASP, 3 showed inefficacy or intolerance at ≥6 months, whereas of the 12 cases of improvement at 2 months after switching, 9 (75%) achieved remission by 12 months after switching. Of the 8 cases with inefficacy at 2 months, 1 (12%) achieved remission at 6 months (Figure 4A).

Of the 18 cases of efficacy at 2 months after switching between mesalazine formulations, 6 showed inefficacy or intolerance thereafter, whereas of the 5 cases of improvement at 2 months, 2 (40%) achieved remission by 6 months. Of the 30 cases that showed inefficacy at 2 months, 2 (6.6%) achieved improvement or remission at 6 months (Figure 4B). 

Two cases of efficacy at 2 months after switching from SASP to mesalazine continued to show efficacy through 12 months after switching. Of the 10 cases that showed inefficacy at 2 months, 1 (10%) achieved remission by 6 months (Figure 4C). Thus, efficacy was mostly observed within 2 months after switching, and 34/43 (79%) retained efficacy through 12 months. 

Among nine patients who became intolerant or in whom treatment became ineffective after initial efficacy at switching, six became intolerant 2 months or more after switching due to adverse events (shown in Table S1). In the case of the other three patients, effectiveness was lost, possibly due to recurrence during natural course of the disease. Details of intolerance for each mesalazine/SASP formulation are shown in Table S1. 

### 3.4. Achievement of Steroid-Free Remission after Switching between Mesalazine Formulations and SASP

**Figure 5:**
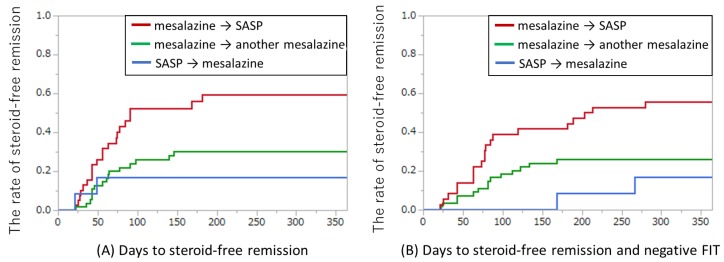
Achievement of steroid-free remission for switching oral mesalazine/SASP formulations. (**A**) Days to steroid-free remission. Red line: from any mesalazine to SASP (*n* = 39); green line: from one mesalazine to another (*n* = 55); blue line: from SASP to any mesalazine (*n* = 12). (**B**) Days to steroid-free remission and negative FIT. Red line: from any mesalazine to SASP (*n* = 36); green line: from one mesalazine to another (*n* = 54); blue line: from SASP to any mesalazine (*n* = 12). Four cases with negative FIT results at switching were excluded. FIT, fecal immunochemical test; SASP: sulfasalazine.

Because more than one-third of our cases received oral and/or topical corticosteroids at the time of switching, the achievement of steroid-free remission was evaluated (Figure 5A). At 12 months after switching, steroid-free remission was achieved in 23/39 (59%) of subjects switching from mesalazine to SASP, in 17/55 (30%) of subjects switching from mesalazine to another mesalazine, and in 2/12 (17%) of subjects switching from SASP to mesalazine. The median (interquartile range) of days to the achievement of steroid-free remission was 56 (39–87), 61 (42–91), and 35 (21–49) days in the groups switching from mesalazine to SASP, from mesalazine to another mesalazine, and from SASP to mesalazine, respectively. Of the 42 cases with steroid-free remission, 35 (83%) achieved it within 100 days. 

In the present study, the FIT was used as a surrogate marker of the achievement of mucosal healing. Figure 5B showed the days to achievement of both steroid-free remission and negative FIT after switching oral mesalazine/SASP formulations. In this analysis, four cases that had negative FIT at switching were excluded. Both steroid-free remission and negative FIT results were achieved in 20/36 (55%) of subjects switching from mesalazine to SASP, 16/54 (29%) of subjects switching from mesalazine to another mesalazine, and 2/12 (17%) of subjects switching from SASP to mesalazine, respectively. Of the 41 patients with steroid-free remission, 38 (92%) achieved both steroid-free remission and FIT negative results. The median (interquartile range) of days to the achievement of both steroid-free remission and FIT negative results was 77 (57–134), 81 (52–117), and 217 (168–266) days in the groups switching from mesalazine to SASP, from mesalazine to another mesalazine, and from SASP to mesalazine, respectively. The time lag between the achievement of steroid-free remission and the achievement of negative FIT results suggests that mucosal healing occurs after the achievement of clinical remission. The endoscopic findings and FIT results from two cases are shown in Figure S3.

## 4. Discussion

The current study demonstrates the efficacy of switching mesalazine/SASP for active UC patients despite the administration of high doses of these agents. Efficacy was observed in 23 (59%) of 39 cases with switching from mesalazine to SASP, 18 (33%) of 55 cases with switching between mesalazine formulations, and 2 (17%) of 12 cases with switching from SASP to mesalazine. The efficacy of switching was generally observed within two months, and most patients who responded to switching achieved steroid-free remission.

Oral mesalazine in its uncoated form is mostly absorbed by the small intestine, with very little active substance reaching the colorectum, and the drug delivery systems of various formulations that are currently available have been optimized to allow the drugs to reach the affected colorectum and minimize systemic absorption [6]. At present, three types of formulation of oral mesalazine are available in Japan: time-dependent release mesalazine (Pentasa), pH-dependent release mesalazine (Asacol), and once-daily MMX mesalazine (Lialda).

The time-dependent formulation consists of mesalazine microspheres encapsulated in an ethylcellulose semipermeable membrane. This structure allows the time- and moisture-dependent release of the active drug, independent of the luminal pH. Mesalazine is theoretically distributed gradually throughout the gastrointestinal tract from the duodenum to the rectum [18]. The pH-dependent formulation encapsulates the active substance in an enteric coat in order to regulate the site of drug release. The coating consists of an enteric resin film designed to dissolve at pH 7, therefore preventing premature disintegration in the stomach and proximal small bowel. Once-daily MMX is a once-daily formulation of mesalazine with a multi-matrix system. Mesalazine is incorporated into a lipophilic matrix, which is in turn dispersed within a hydrophilic matrix. The tablet is enterically coated and dissolves in the terminal ileum at approximately pH 6.8. The hydrophilic matrix is exposed to intestinal fluid and swells to form a viscous gel mass. This viscous gel potentiates slow diffusion of the active substance from the tablet core, thereby enabling a slow, controlled release of mesalazine throughout the entire length of the colon [19].

Although several reports have compared the efficacy among mesalazine formulations, few have compared the efficacy at equivalent doses. Gibson et al. [9] performed a randomized double-blind trial that showed that 3 g/day of pH-dependent mesalazine and 3 g/day of time-dependent mesalazine achieved comparable rates of clinical remission after 8 weeks. In contrast, Ito et al. [20] reported the noninferiority of 2.4 g/day of pH-dependent mesalazine to 2.25 g/day of time-dependent mesalazine. They also reported that 2.4 g/day of pH-dependent mesalazine was significantly more effective than a placebo in patients with proctitis-type disease, but 2.25 g/day of time-dependent mesalazine was not. Prantera et al. [21] compared 2.4 g/day of once-daily MMX mesalazine to 2.4 g/day of pH-dependent mesalazine as maintenance therapy in 331 patients with left-side UC. After 12 months, the two formulations were comparable in maintaining clinical and endoscopic remission based on a clinician’s assessment (60.9 and 61.7%, respectively). Thus, the most desirable formulation is currently undetermined in terms of efficacy and safety.

Studies that have shown the efficacy of switching between mesalazine formulations with equivalent dose are also scarce. For example, Kawashima et al. [22] reported the efficacy of 3.6 g/day of pH-dependent mesalazine for UC patients with mild to moderate activity who show resistance to 2.25 g/day of time-dependent mesalazine. Wong et al. [23] reported that 9 UC patients with endoscopic evidence of active disease, despite treatment with 2.4 g/day of pH-dependent mesalazine switched to 4.0 g/day of time-dependent mesalazine. Definite conclusions cannot be elicited from such unbalanced switching conditions.

In the present study, switching was performed between the maximum doses of each mesalazine formulation. The efficacy at 2 months after switching to time-dependent mesalazine, pH-dependent mesalazine, and once-daily MMX mesalazine was 2/7 (29%) of cases, 6/22 (27%) of cases, and 10/26 (38%) of cases, respectively. Switching to MMX mesalazine may thus be slightly more effective than switching to time-dependent mesalazine or pH-dependent mesalazine, although there was no significant difference. However, intolerance to MMX mesalazine was more frequent than it was to other formulations (0% for Pentasa, 5% for Asacol, and 23% for Lialda). The adverse events associated with switching to once-daily MMX mesalazine included abdominal discomfort, rash, cough, renal function disorder, and liver function disorder. Both these favorable and unfavorable effects may be due in part to the dose of MMX mesalazine (4.8 g/day) being higher than that of other formulations (Pentasa: 4 g/day; Asacol: 3.6 g/day). It is also possible that a special coating associated with MMX technology was responsible.

Regarding switching from one mesalazine to SASP, Yoshino et al. [11] reported the efficacy of switching to SASP (*n* = 25) or adding SASP (*n* = 11) in 36 UC patients refractory to mesalazine (>4 g/day of time-dependent mesalazine or >3.6 g/day of pH-dependent mesalazine), with 25 achieving clinical remission. In the current study, switching from mesalazine to SASP was effective in 59% of patients. These results were in line with those of previous reports [11,24]. The usefulness of SASP for inducing and maintaining remission in UC patients is well established [25,26]. However, because less mesalazine is included in SASP than in the same dose of other mesalazine formulations, the efficacy of SASP might also be exerted by other mechanisms than the release of mesalazine in the colon. Indeed, Fujiwara et al. reported that SASP might inhibit the T-cell-dependent antibody response partly through the suppression of IL-2 production [27,28]. Matastic et al. further showed that SASP inhibited the maturation of human dendritic cells [29], and Rodenburg et al. indicated that SASP inhibited TNF-alpha expression in macrophages by inducing apoptosis [30].

However, adverse events are more frequent when using SASP than when using other mesalazine formulations. A meta-analysis demonstrated that 29% of patients treated with SASP experienced adverse events compared with 14% of those using other mesalazine agents (relative risk = 0.48, 95% confidence interval: 0.36–0.63) [4]. Both non-dose-dependent and dose-dependent adverse events have been reported in SASP. Dose-dependent adverse events include headache, nausea, and fatigue. When dose-dependent adverse events occur, dose reduction of formulations is a useful method of resolving the issue. Therefore, in the present study, the dose of SASP was gradually increased at switching when adverse events of SASP were of concern. However, despite this approach, 25% of patients who switched to SASP showed intolerance. Based on the results of our study, time-dependent mesalazine or pH-dependent mesalazine should be considered first, as these formulations cause fewer adverse events than SASP or MMX mesalazine. When mild activity persists despite a sufficient dose of mesalazine formulation, switching to SASP should be considered.

One strength associated with this study is its inclusion of highly refractory patients. Indeed, 59 patients were using immunomodulators, tacrolimus, or anti-TNF agents. Although the characteristics of our patients differed from each other in this context, our results confirmed that switching of mesalazine/SASP was effective for a variety of UC patients, regardless of concomitant therapy. Of these refractory patients, 20 (33%) achieved remission simply by mesalamine/SASP switching. However, more potent and costly therapies with a possible risk of adverse events were required in the remaining 39 switch-ineffective cases (e.g., 13 received new biologics, and 3 received tofacitinib). Safer and less costly therapies, including *Escherichia coli* Nissle 1917 [31] and IBD98-M [32], could be applicable in the treatment of these patients in the future.

Another strength is the evaluation of the long-term effect and steroid-free remission rate following switching of mesalazine/SASP, as most previous reports have evaluated only the short-term clinical response. As UC is a long-lasting disease, only short-term efficacy with therapeutics is insufficient. Furthermore, because most patients who were candidates for switching the mesalazine/SASP formulation had mild activity, the improvement in symptoms might have been a “placebo effect.” Therefore, to verify the efficacy of switching in this study, the FIT was used as a biomarker of the achievement of mucosal healing. These meticulous evaluations improved the reliability of this study despite the retrospective nature of the analysis.

Several limitations associated with the present study warrant mention. First, this was a retrospective study with a relatively small number of patients conducted in a single-center setting. Second, while a considerable number of patients might use generic versions of mesalazine/SASP, the difference in efficacy between the generic versions and originators could not be determined.

In conclusion, we observed the efficacy of switching of mesalazine/SASP for the induction of remission in patients with UC, particularly in cases switching from mesalazine to SASP. Switching mesalazine/SASP may be a viable therapeutic option for UC patients with a relatively low clinical activity, even after the administration of high doses of these agents.

## Figures and Tables

**Table 1:** Characteristics of the study population.

Patients	88
**Gender**	
Male/Female	48/40
**Location of disease**	
Pancolitis/Left-sided/Proctitis	58/26/4
Median (IQR) age at diagnosis, years	30 (18–44)
Median (IQR) disease duration, years	10 (7–16)
Median (IQR) age at the switching, years	38 (26–50)
**Switching of mesalazine/SASP formulations**	106
Median (IQR) PRO2	2 (2–3)
**Formulations of mesalazine or SASP before switch**	
Time-dependent/pH-dependent/once-daily MMX/SASP	46/48/0/12
**Formulations of mesalazine or SASP after switch**	
Time-dependent/pH-dependent/once-daily MMX/SASP	11/27/29/39
**Concomitant drugs**	
Oral corticosteroid	22
Topical corticosteroid	15
Immun omodulator	51
Tacrolimus	17
Anti-TNFα agent	4

IQR: interquartile range; PRO2: two-item patient-reported outcome; SASP: sulfasalazine; MMX: multi-matrix system.

## Supplementary Materials

The following are available online at https://www.mdpi.com/2077-0383/8/12/2109/s1. Figure S1: Relation between PRO2 and Lichtiger clinical activity index in patients who underwent switch. PRO2: two-item patient-reported outcome, Figure S2: Effectiveness at two months after switching each type of mesalazine/SASP formulations. (A) from each type of mesalazine to SASP (*n* = 39), (B) from each type of mesalazine to another (*n* = 55), and (C) from SASP to each type of mesalazine (*n* = 12). SASP, sulfasalazine; MMX, multi-matrix Figure S3: Endoscopic findings and FIT results in 2 cases. FIT: fecal immunochemical test. Table S1: Details of intolerance.
