# Supplementary material for: Switching between Three Types of Mesalazine Formulation and Sulfasalazine in Patients with Active Ulcerative Colitis Who Have Already Received High-Dose Treatment with These Agents
Source: J Clin Med. 2019 Dec 2;8(12):2109. doi: 10.3390/jcm8122109 (PMC6970226; doi:10.3390/jcm8122109)
Supplement: Supplementary file 1 [file jcm-08-02109-s001.docx]

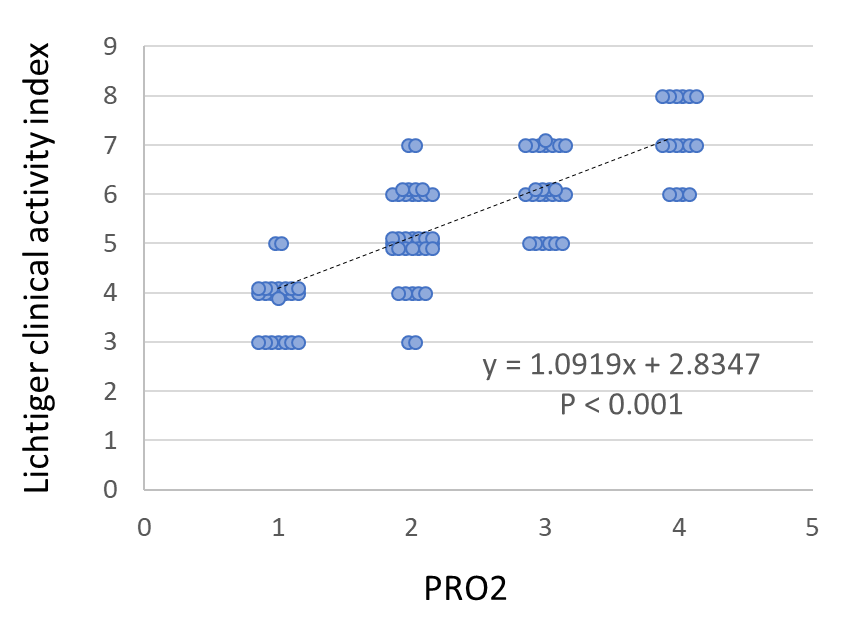


**Figure S1.** Relation between PRO2 and Lichtiger clinical activity index in patients who underwent switch. PRO2, two-item patient-reported outcome

**
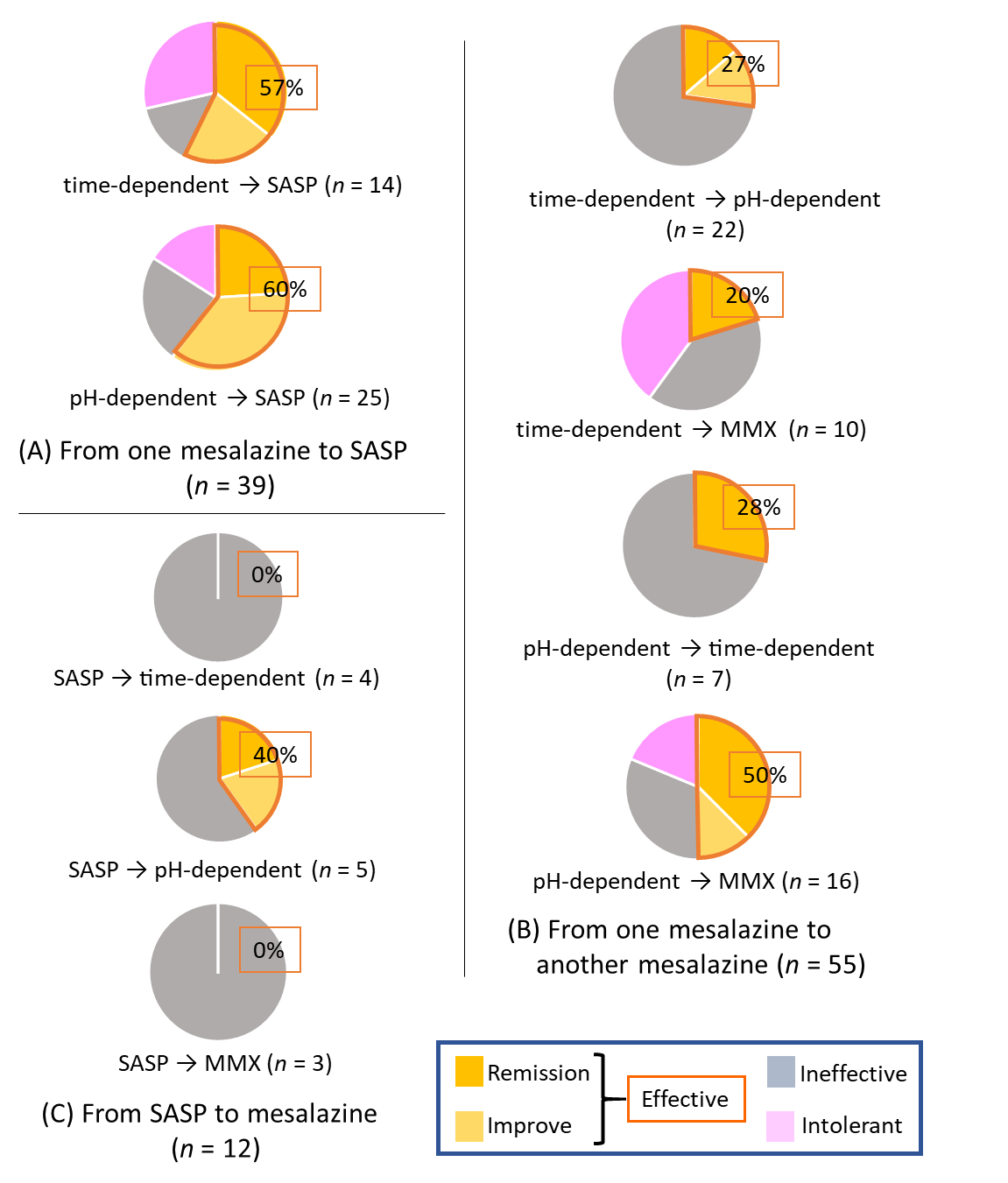
**

**Figure S2.** Effectiveness at two months after switching each type of mesalazine/SASP formulations. (**A**) from each type of mesalazine to SASP (*n* = 39), (**B**) from each type of mesalazine to another (*n* = 55), and (**C**) from SASP to each type of mesalazine (*n* = 12). SASP, sulfasalazine; MMX, multi-matrix system

**Table S1.** Details of intolerance

| **Timing of the Appearance** | | |
| --- | --- | --- |
| 0–2 months (*n* = 14) | 2–6 months (*n* = 5) | 6–12 months (*n* = 2) |
| **SASP (*n* = 7)**  quincke’s edema*, rash, and headache (*n* = 1)  nausea (*n* = 2)  rash (*n* = 2)  fever (*n* = 1)  headache (*n* = 1)  **MMX (*n* = 7)**  epigastralgia (*n* = 2)  rash (*n* = 1)  dyspnea (*n* = 1)  cough (*n* = 1)  liver function disorder (*n* = 1)  fever (*n* = 1) | **SASP (*n* = 2)**  epigastralgia (*n* = 1)  change of urine color (*n* = 1)  **MMX (*n* = 3)**  renal disorder** (*n* = 1)  dysphagia (*n* = 1)  epigastric distress (*n* = 1) | **SASP (*n* = 1)**  general fatigue  **time-dependent** **(*n* = 1)**  smell of urine |

Total, *n* = 21 (SASP, *n* = 10; time-dependent mesalazine, *n* =1; once-daily MMX mesalazine, *n* =10). *quinke’s edema: angioedema, a form of localized swelling of the deeper layers of the skin and fatty tissues beneath the skin. **renal disorder: a rise in the serum creatinine level

**
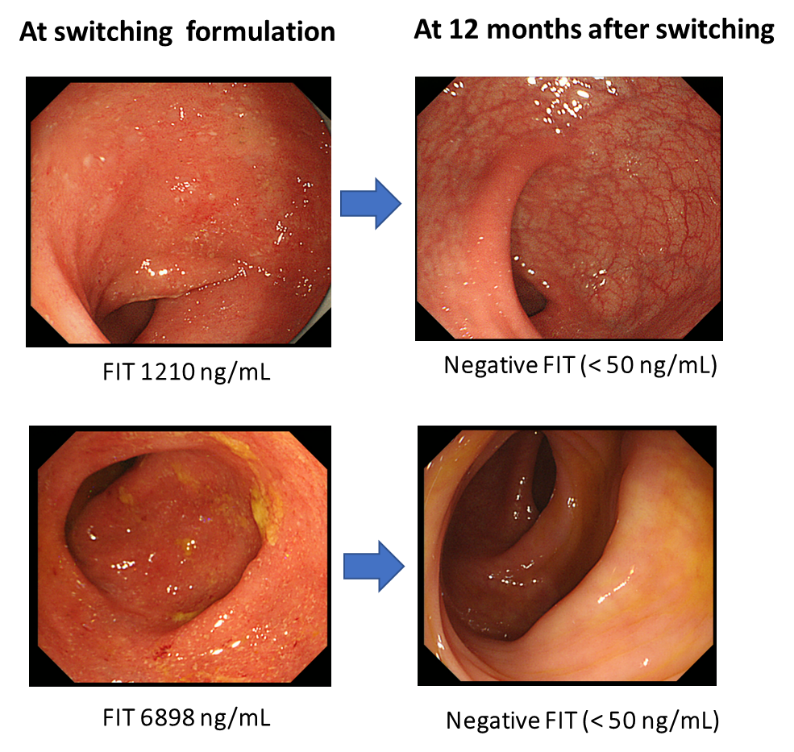
**

**Figure S3**. Endoscopic findings and FIT results in 2 cases. FIT, fecal immunochemical test
